# Reovirus mutant jin-3 exhibits lytic and immune-stimulatory effects in preclinical human prostate cancer models

**DOI:** 10.1038/s41417-021-00360-2

**Published:** 2021-06-16

**Authors:** Arjanneke F. van de Merbel, Geertje van der Horst, Maaike H. van der Mark, Selas T. F. Bots, Diana J. M. van den Wollenberg, Corrina M. A. de Ridder, Debra Stuurman, Tilly Aalders, Sigrun Erkens-Schulz, Nadine van Montfoort, Wouter R. Karthaus, Niven Mehra, Minke Smits, Jack A. Schalken, Wytske M. van Weerden, Rob C. Hoeben, Gabri van der Pluijm

**Affiliations:** 1 Department of Urology, Leiden University Medical Center, Leiden, The Netherlands; 2 Department of Cell and Chemical Biology, Leiden University Medical Center, Leiden, The Netherlands; 3 Department of Experimental Urology, Erasmus Medical Center, Rotterdam, The Netherlands; 4 Department of Urology, Radboud University Medical Center, Nijmegen, The Netherlands; 5 Department of Medical Oncology, Leiden University Medical Center, Leiden, The Netherlands; 6 Human Pathology and Pathogenesis Program, Memorial Sloan Kettering Cancer Center, New York, NY USA; 7 Department of Medical Oncology, Radboud University Medical Center, Nijmegen, The Netherlands

**Keywords:** Prostate cancer, Cancer models

## Abstract

Treatment of castration-resistant prostate cancer remains a challenging clinical problem. Despite the promising effects of immunotherapy in other solid cancers, prostate cancer has remained largely unresponsive. Oncolytic viruses represent a promising therapeutic avenue, as oncolytic virus treatment combines tumour cell lysis with activation of the immune system and mounting of effective anti-tumour responses. Mammalian *Orthoreoviruses* are non-pathogenic human viruses with a preference of lytic replication in human tumour cells. In this study, we evaluated the oncolytic efficacy of the bioselected oncolytic reovirus mutant *jin-3* in multiple human prostate cancer models. The *jin-3* reovirus displayed efficient infection, replication, and anti-cancer responses in 2D and 3D prostate cancer models, as well as in ex vivo cultured human tumour slices. In addition, the *jin-3* reovirus markedly reduced the viability and growth of human cancer cell lines and patient-derived xenografts. The infection induced the expression of mediators of immunogenic cell death, interferon-stimulated genes, and inflammatory cytokines. Taken together, our data demonstrate that the reovirus mutant *jin-3* displays tumour tropism, and induces potent oncolytic and immunomodulatory responses in human prostate cancer models. Therefore, *jin-3* reovirus represents an attractive candidate for further development as oncolytic agent for treatment of patients with aggressive localised or advanced prostate cancer.

## Introduction

Prostate carcinoma is the second most common cancer and the fifth leading cause of cancer-related death in men worldwide [1]. The development of therapy resistance and incurable metastatic disease represents major clinical problems.

Immunotherapy has emerged as a viable and attractive strategy for the treatment of different solid cancers [2]. Despite the success of immunotherapeutic approaches in various cancers, prostate cancer has remained largely unresponsive for single-agent immune therapies, including cancer vaccines and immune checkpoint inhibitors [3]. Accumulating evidence suggests that prostate cancer cells escape from immune surveillance by creating an immune-suppressive and immune-exclusive tumour microenvironment [4]. This immunosuppressive barrier impairs the generation and maintenance of a clinically desired anti-tumour immune response. Treatment modalities that overcome this immunosuppressive state could represent a promising option for prostate cancer.

Oncolytic viruses specifically infect, replicate, and lyse malignant tumour cells, while minimising harm to normal cells. Moreover, oncolytic viruses have the ability to promote adaptive and innate immune responses upon infection and killing of cancer cells, e.g., mediated by the release of danger-associated molecular patterns (DAMPs) like high mobility group box 1 (HMGB1) [5–8]. Previously, we have demonstrated that the oncolytic potency of mammalian *Orthoreoviruses* can be enhanced by natural selection and genetic modification [9, 10]. Reoviruses are double-stranded RNA viruses and have not been associated with severe disease in humans [11]. Wild-type reovirus type 3 Dearing (T3D) has oncolytic properties in a variety of tumour types. It binds to a cancer cell by interaction of viral spike protein Sigma-1 to sialic acids and to junction adhesion molecule A (JAM-A) [10, 12, 13]. However, JAM-A expression is often reduced in solid cancers and this correlates with a poor survival and a worse prognosis [14]. Mutant reoviruses with enhanced tumour tropism, that can also infect cancer cells independently of JAM-A (i.e., via negatively charged sialic acids) represent a promising treatment modality [10]. Our group has generated spontaneous reovirus mutants (i.e., *jin-1*, *−2*, and *−3*) with extended tropism. These reovirus mutants are able to infect a wide range of cell lines that normally resist wild-type reovirus T3D infection [10]. In the absence of JAM-A, these reovirus mutants depend on negatively charged sialic acids on the cell surface for infection.

In this study, the direct oncolytic and indirect immunomodulatory effects of *jin-3* reovirus were determined in state-of-the-art preclinical prostate cancer models, including monolayer and three-dimensional cell cultures, ex vivo cultured human prostate cancer tissue slices, and cell line- and patient-derived prostate cancer xenograft models in vivo [15].

## Material and methods

### Virus production

Wild-type T3D reovirus strain R124 was plaque purified from the wild-type reovirus T3D (ATCC, Manassas, VA, United States) on HER911 cells [10, 16]. Reovirus mutant *jin-3* was isolated from JAM-A-deficient U118MG cells after passaging of the wild-type T3D strain R124 [10]. Both R124 and *jin-3* reoviruses were propagated, purified, and titrated on human HER911 cells as described [10]. Cell lines were propagated for no >6 months or 30 passages after resuscitation from stocks. All cell lines were frequently tested for *Mycoplasma* infection, using a *Mycoplasma*-specific polymerase chain reaction (PCR).

### Two- and three-dimensional prostate cancer cultures

Human prostate cancer cell lines PC-3M-Pro4luc2, DU145, and 22Rv1 were cultured in monolayers (Table S1). Three-dimensional cultures were generated from a previously established three-dimensional prostate cancer model from bone metastasis material (MSK-PCa1), or generated from newly established patient-derived xenograft (PDX) models from prostate cancer bone and liver metastases biopsies (NM78 and NM72) [17, 18].

Three-dimensional cultures of prostate cancer bone and liver metastases were maintained, as previously described [17, 18].

### Viability assays

For cell lines, 1500 cells were seeded per well in a 96-well plate. After 24 h, cells were exposed to oncolytic reovirus at a multiplicity of infection (MOI) of 0.01–0.1–1–10 and 100 plaque forming units (p.f.u.)/cell. After 24 h, the medium was refreshed. After 6 days, the viability of the cells was assessed by performing MTS assays [19]. Three-dimensional prostate cancer cultures were treated with oncolytic reovirus for 3, 7, and 10 days. Changes in viability were assessed by the Cell Titre Glo assay, according to the manufacturer’s protocol (Promega, Madison, WI, United States).

### FACS analyses

Flow cytometry was performed with LSRII (BD Biosciences, Franklin Lakes, NJ, United States) and analysed with FCS express software after staining the cells with JAM-A antibody. (Table S2)

### Sigma-3 immunocytochemistry

A total of 20,000 prostate cancer cells were seeded in eight-well chamber slides (ThermoFisher Scientific, Waltham, MA, United States). After 24 h, the cells were exposed to oncolytic reovirus After 1, 2, and 3 days of post exposure, Sigma-3 was visualised by immunofluorescence (Table S2) and confocal microscopy.

### Generation of prostate cancer patient-derived xenograft models and ex vivo tumour tissue slice culture

Prostate cancer tissue was obtained via either transurethral resection of the prostate (prostatectomy) or needle biopsies after informed consent (Pronet p05.85 and RBUT-ID-PROSTAAT-151; Table S3).

In order to establish new prostate cancer PDX models, tumour pieces were implanted subcutaneously in adult male immunodeficient mice. All animal experiments were performed after approval by the Animal Welfare Committee of the Leiden University Medical Center in accordance with the Dutch Act on Animal experimentation and EU Directive 2010/63/EU (project licences from Central Authority for Scientific Procedures on Animals (CCD): AVD1160020173725 and AVD1160020187004). Established and well-characterised prostate cancer PDX models were propagated as described (project licence AVD101002017867) [20] (Table S4). Tumour growth was monitored by calliper measurements. All mice were housed under sterile conditions in accordance with Dutch guidelines.

Prostate cancer tissue was sliced and cultured, as previously described [19]. Slices were exposed to 10^8^ p.f.u./ml *jin-3* reovirus. Three days post exposure, the tissues were fixed with 4% PFA and processed for histology.

### Histology and Sigma-3 scoring

H&E and immunofluorescent stainings were executed, as previously described (Table S2) [19]. H&E-stained sections were analysed and Sigma-3-stained tumour cells were scored with the Pannoramic MIDI slide scanner (3DHISTECH, Budapest, Hungary). All fluorescently stained cells or tissue sections were visualised by confocal microscopy (63× magnification, resolution 1024 × 1024; Leica SP8, Wetzlar, Germany). Slide scans from Sigma-3 stainings were scored. Four sections were scored per condition for positive Sigma-3 staining by two independent reviewers (Fig. S1).

### Real-time quantitative polymerase chain reaction (RT-qPCR)

Cells were seeded in six-well plates and exposed to oncolytic reovirus for 6, 24, and 48 h. Total RNA was isolated according to the manufacturer’s protocol (Nucleospin RNA kit Macherey-Nagel, Düren, Germany). cDNA was generated by using random primers (Promega, Madison, WI, United States) and RT-qPCR was performed with GoTaq Mastermix (Promega, Madison, WI, United States), according to the manufacturer’s protocol in technical duplicates and biological triplicates (Promega, Madison, WI, United States). Gene expression was normalised to *GAPDH* expression. The sequences of the PCR primers used for the quantitation of cellular transcripts, viral RNA, and the detection of mycoplasma contamination can be found in Table S5.

### High mobility group box 1 release

Cells were exposed to oncolytic reovirus with a MOI of 10. At 48 h post exposure, conditioned medium was collected. HMBG1 release was measured by performing an ELISA, according to the manufacturer’s protocol (IBL International, Hamburg, Germany).

### Administration of *jin-3* reovirus in vivo

Subcutaneous PC-3M-Pro4luc2 tumours were generated in male NSG mice (AVD1160020173725 and AVD1160020187004) [19]. When tumours reached a volume of 0.11 cm^3^, intra-tumoural administration with 10^8^ p.f.u. *jin-3* reovirus in 10 μl PBS was initiated twice a week (*n* = 6 per group). For sample size calculations and in/exclusion criteria, see Supplementary Information. Tumour growth was monitored by bioluminescence imaging [21]. Tumour pieces of PDX model PCa-15.01 were implanted subcutaneously in the flank of male NSG mice (AVD1160020173725 and AVD1160020187004). When tumours reached a volume of 0.11 cm^3^, mice were randomised based on body weight, tumour burden, and/or tumour size. Subsequently, tumours were treated by intra-tumoural injections of 10^8^ p.f.u. *jin-3* reovirus (*n* = 10 per group). Tumour growth was assessed by calliper measurements.

### Statistical analyses

Statistical analyses were performed by using GraphPad Prism 8.0. One-way ANOVA was performed for viability experiments. All in vitro experiments were repeated at least twice. Data are represented as mean ± standard error of the mean (SEM). Two-way ANOVA and two-sided *t* tests were performed on data from in vivo experiments. *p < 0.05, **p < 0.01, ***p < 0.001, and ****p < 0.0001.

## Results

### Direct oncolytic effects of reovirus mutant *jin-3* reovirus in vitro

**Fig. 1:**
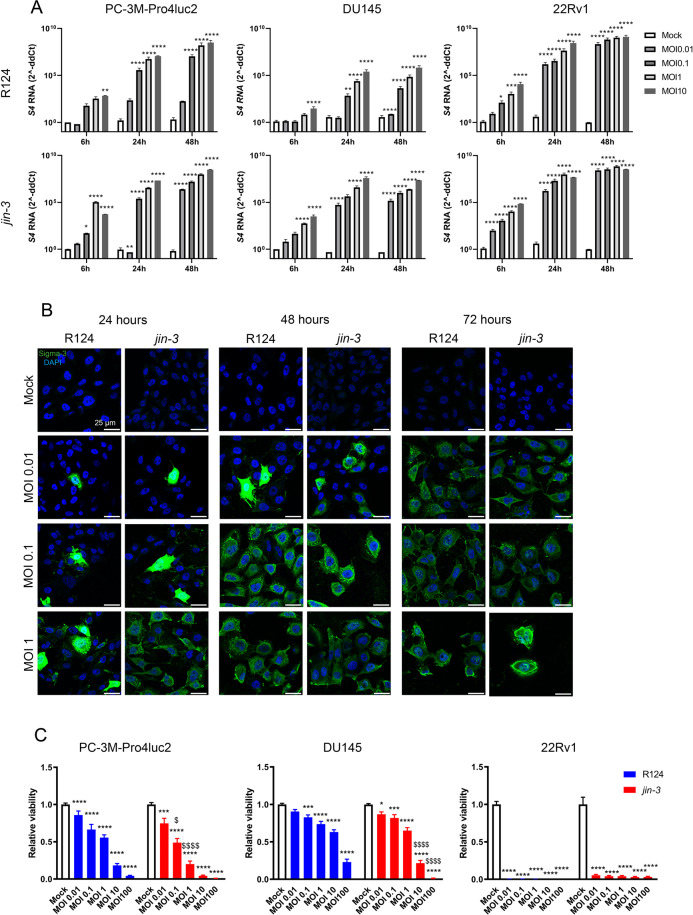
Infection, replication, and oncolytic effects of reovirus *jin-3* mutant versus wild-type R124 parental reovirus in prostate cancer cell lines in vitro. **A** Detection of viral transcripts (capsid protein S4) by RT-qPCR indicated dose- and time-dependent infection and replication of R124 reovirus (upper row) and *jin-3* reovirus (bottom row) in human prostate cancer cells PC-3M-Pro4luc2, DU145, and 22Rv1. Gene expression is represented as 2^−ddCt^ ± standard error of the mean (SEM), *N* = 3. Two-way ANOVA. MOI = multiplicity of infection. *p < 0.05, **p < 0.01, ***p < 0.001, ****p < 0.0001. * mock versus reovirus infection. **B** Confocal microscopy for Sigma-3 viral capsid protein (green) in PC-3M-Pro4luc2 cells indicated dose-dependent and time-dependent immunofluorescent localisation of Sigma-3 viral capsid protein (green) in PC-3M-Pro4luc2 prostate cancer cells that were exposed to R124 wild-type and *jin-3* reoviruses. Green Sigma-3 viral capsid protein, blue DAPI (nuclei). Magnification is 63×, scale bar = 25 μm. **C** Dose-dependent killing of human PC-3M-Pro4luc2, DU145, and 22Rv1 prostate cancer cell lines (cell viability) upon exposure of these cells with R124 wild-type and *jin-3* reoviruses for 6 days. *p < 0.05, ***p < 0.001, ****p < 0.0001, ^$^*p* < 0.05,^$$$$^*p* < 0.0001. * mock versus reovirus infection, ^$^ R124 versus *jin-3*. Mean ± standard error of the mean (SEM), *N* = 3. Two-way ANOVA. MOI multiplicity of infection.

Prostate cancer cells PC-3M-Pro4luc2, DU145, and 22Rv1 were exposed to R124 and *jin-3* reoviruses, and viral infection was monitored by RT-qPCR and confocal microscopy. Dose-dependent and time-dependent reoviral infection was observed upon exposure to R124 and *jin-3* reovirus (Fig. 1A, B). Next, viability assays were performed in AR-negative prostate cancer cell lines PC-3M-Pro4luc2 and DU145 cells and AR-positive 22Rv1 cells. *Jin-3* reovirus significantly reduced the viability of all prostate cancer cell lines (Fig. 1B). 22Rv1 cells were extremely sensitive to reovirus exposure, whereas AR-negative cell lines DU145 and PC-3M-Pro4luc2 displayed a dose-dependent decrease in viability after exposure to *jin-3* (Fig. 1B). When compared to PC-3M-Pro4luc2 and DU145 cells exposed to R124 reovirus, the viability of *jin-3* reovirus exposed PC-3M-Pro4luc2 and DU145 cells was significantly reduced (^$^*p* < 0.05 and ^$$$$^*p* < 0.0001 MOI1 and MOI10 in PC-3M-Pro4luc2, and ^$$$$^*p* < 0.0001 MOI10 and MOI100 in DU145; Fig. 1C). FACS analyses revealed that 98%, 97%, and 89% of PC-3M-Pro4luc2, DU145, and 22Rv1 cells expressed JAM-A protein, respectively.

**Fig. 2:**
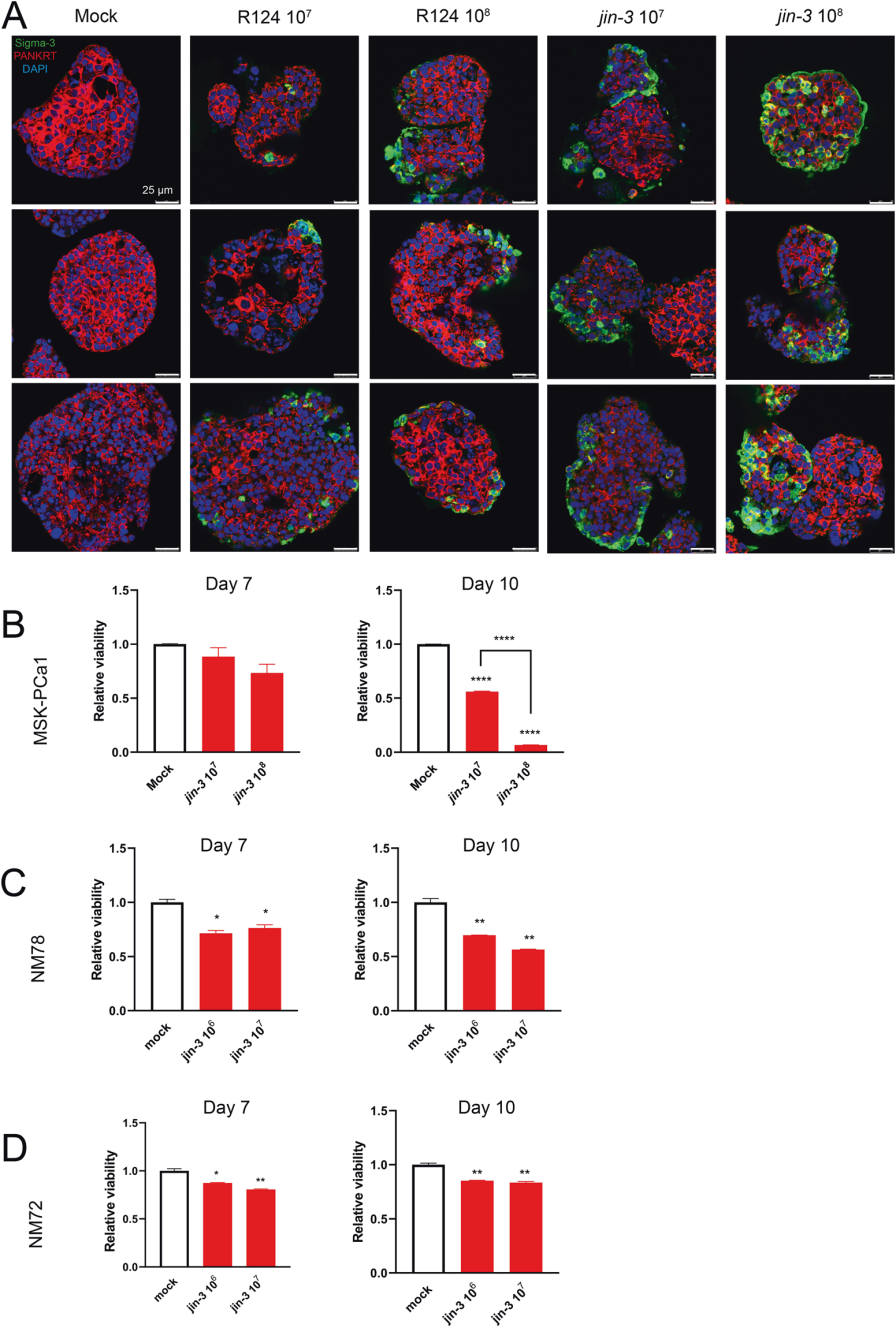
Reovirus infection and replication in three-dimensional cultures of human prostate cancer. **A** Three-dimensional cultures from MSK-PCa1 prostate cancer cells, derived from a bone metastasis [17] were exposed to 10^7^ and 10^8^ plaque forming units (p.f.u.) of R124 or *jin-3* reovirus. After 3 days, staining for reovirus (Sigma-3) was observed in the outer cell layers of the 3D cultures, indicating active viral infection and replication. Green Sigma-3 (viral protein), red pan-cytokeratin (tumour cells), blue DAPI (nuclei). Magnification is 63×, scale bar = 25 μm. **B-D** Three-dimensional cultures of metastatic human prostate cancer were generated and exposed to *jin-3* reovirus for 7 and 10 days. Viability assays indicated a significant reduction of cellular viability after exposure to *jin-3*. Mean ± standard error of the mean (SEM), *p < 0.05, **p < 0.01, ****p < 0.0001. One-way ANOVA.

Treatment of three-dimensional cultures of bone metastasis derived MSK-PCa1 cells [17] with oncolytic reovirus revealed a dose-dependent infection and viral replication (Fig. 2A). In addition, treatment with *jin-3* reovirus significantly decreased the viability of three-dimensional cultures of prostate cancer bone and liver metastases (MSK-PCa1, NM78, and NM72) after 7 and 10 days (*p < 0.05, **p < 0.01, ****p < 0.0001, Fig. 2B–D, respectively).

### Reovirus infection and replication in ex vivo cultured prostate cancer tissue slices

**Fig. 3:**
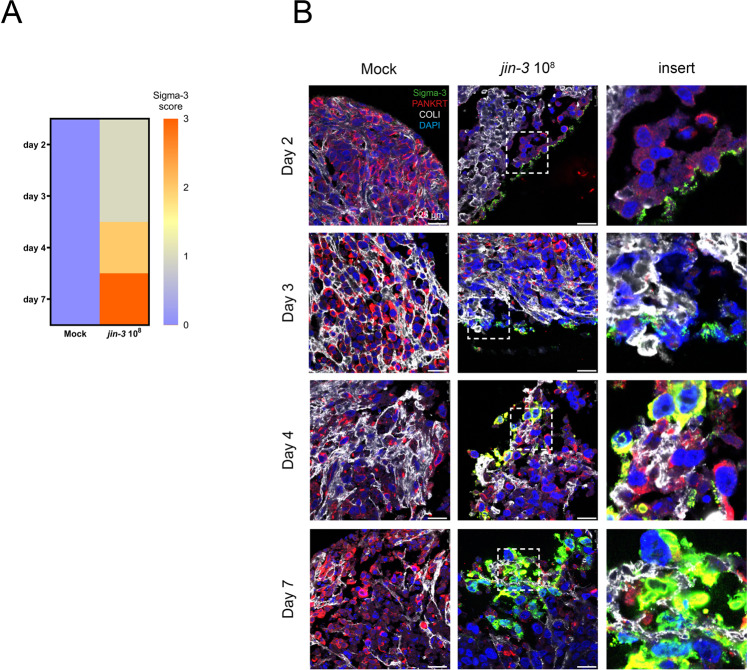
*jin-3* reovirus infection in ex vivo cultured tumour tissue slices from human prostate cancer cell line-derived xenografts. **A** Prostate cancer tissue slices from PC-3M-Pro4luc2 tumours were exposed to 10^8^ p.f.u. *jin-3* reovirus for 2, 3, 4, or 7 days. Scoring of Sigma-3 viral protein indicated a time-dependent increase in Sigma-3 score. **B** Viral infection and replication in ex vivo cultured tissue slices with reovirus. Green Sigma-3 (viral protein), red pan-cytokeratin (tumour cells), blue DAPI (nuclei). Magnification is 63×, scale bar = 25 μM.

Prostate cancer tissue slices were generated from explanted PC-3M-Pro4luc2 tumours and infected with *jin-3* reovirus for 2, 3, 4, and 7 days. Exposure to *jin-3* reovirus resulted in a time-dependent increase in Sigma-3 score indicating viral infection and replication (Fig. S1 and Fig. 3A, B).

**Fig. 4:**
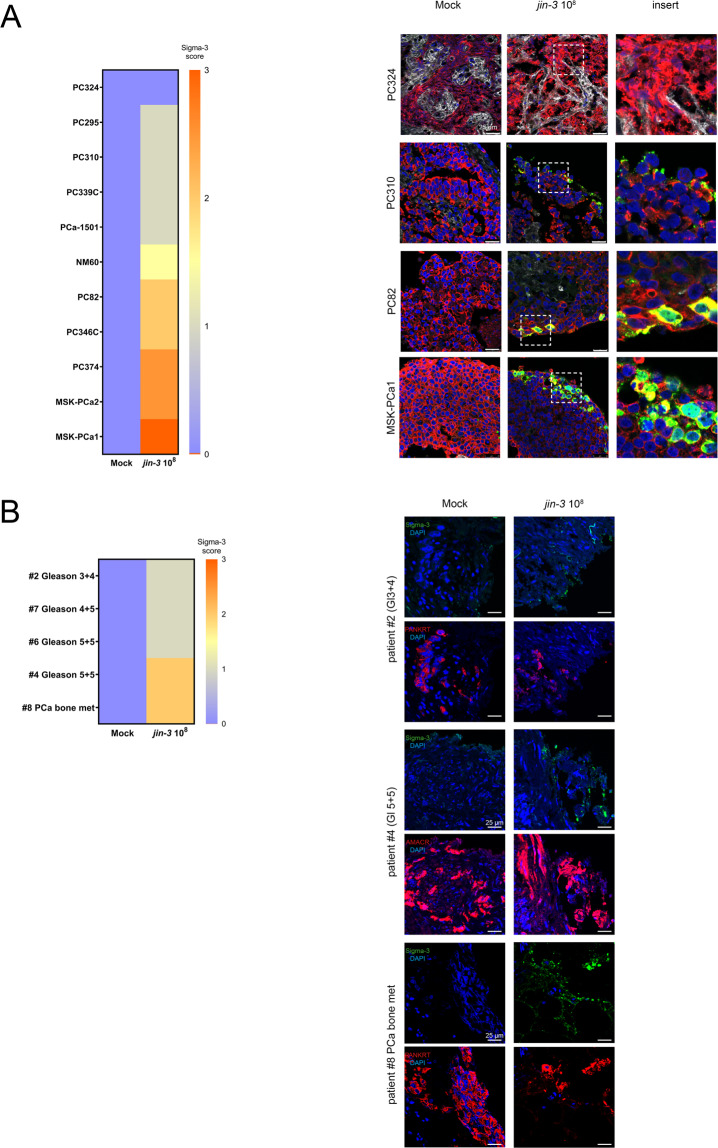
*jin-3* reovirus infection in prostate cancer tissue slices derived from patient-derived xenografts (PDX) and primary prostate cancer. **A** Scoring of Sigma-3 viral protein in ex vivo infected human prostate cancer tissue slices indicated heterogeneous response to exposure to 10^8^ p.f.u. *jin-3* reovirus. **B** Scoring of Sigma-3 viral protein in ex vivo infected primary prostate cancer material with 10^8^ *jin-3* reovirus for 4 days. Green viral capsid protein Sigma-3, red pan-cytokeratin or AMACR (tumour cell markers), blue DAPI (nuclei). Magnification is 63×, scale bar = 25 μm .

Next, tumour tissue slices were generated from novel and previously established PDX models [20, 22, 23] followed by exposure to *jin-3* reovirus for 3 days (Fig. 4A). For the duration of the ex vivo tissue culture experiment, Sigma-3 staining was observed in 91% (10/11) of the PDX models in the *jin-3* reovirus-treated group (Fig. 4A and Fig. S2). JAM-A protein expression was observed in all PDX tumours, but the subcellular localisation of JAM-A protein varies per patient-derived tumour (Fig. S3A). Reovirus infection was observed in both AR-positive and AR-negative PDX models (Fig. S3B). In ex vivo cultured tissue slices from patient biopsy material, Sigma-3 staining was observed in all prostate cancer biopsies after *jin-3* reovirus exposure for 3 days (Fig. 4B and Fig. S4).

### Reovirus mutant *jin-3* induces oncolysis in prostate cancer xenografts in vivo

**Fig. 5:**
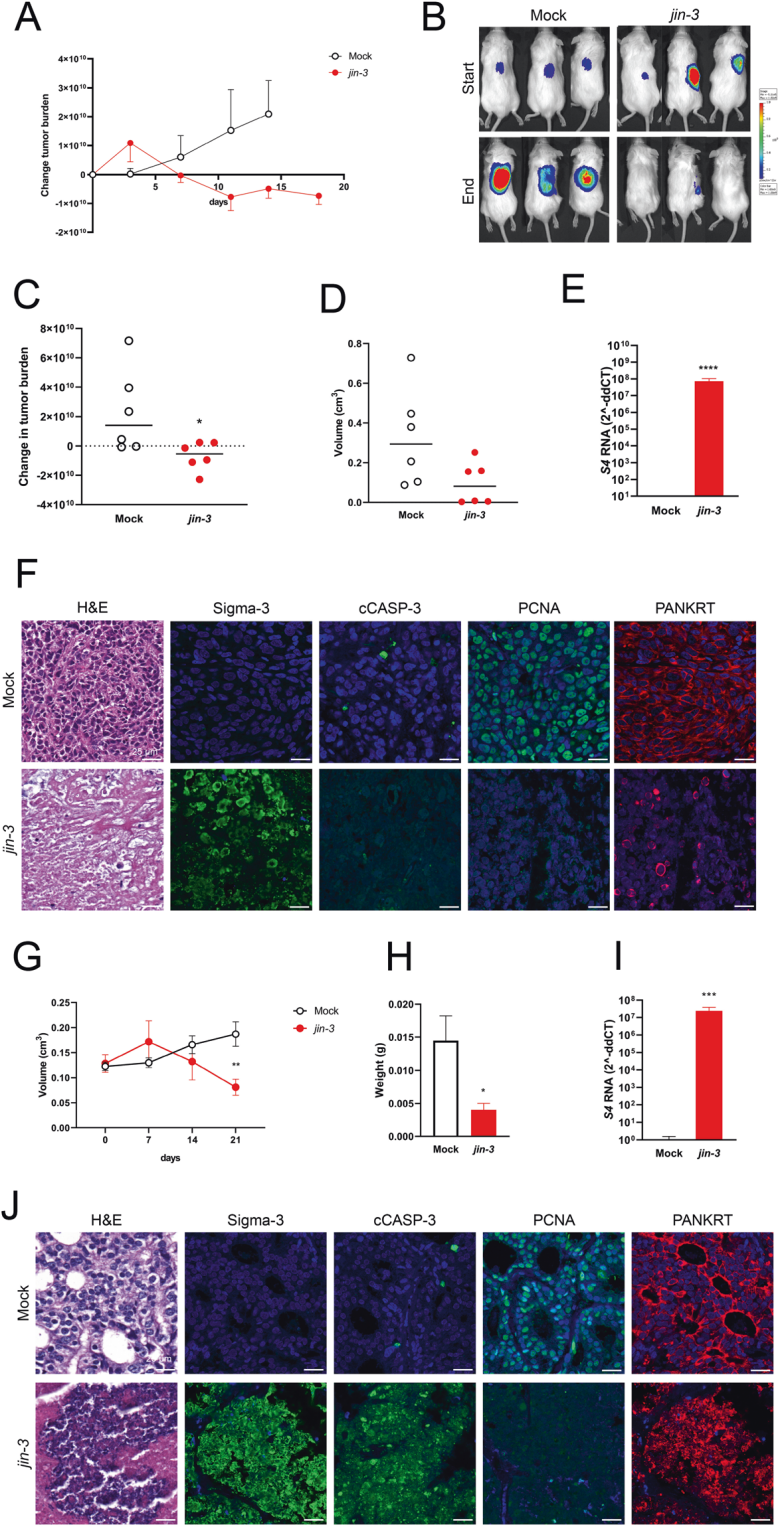
Direct oncolytic effects of *jin-3* reovirus in xenograft models of human prostate cancer models. The effect of intra-tumoural administration of *jin-3* in subcutaneously growing human prostate tumours from cell line-derived xenograft (CDX) PC-3M-Pro4luc2 (**A**–**F)** and patient-derived xenograft (PDX) model PCa-15.01 (**G**–**J**). **A** Effect of *jin-3* reovirus administration on total tumour burden was measured by whole-body bioluminescent reporter imaging (BLI) of firefly-luciferase2 expressing PC-3M-Pro4luc2 cells (*n* = 6 per group). **B** Whole-body bioluminescent optical imaging (BLI) at the start and end of the experiment. **C** Change of tumour burden (BLI) was significantly reduced in *jin-3*-treated tumours (*p* < 0.05). **D** Tumour volume (calliper measurements) was reduced after *jin-3* administration. **E** *S4* RNA expression, indicative of viral Sigma-3 gene expression, was observed in PC-3M-Pro4luc2 tumours treated with *jin-3* reovirus at day 21 (*p* < 0.0001). **F** Histological evaluation depicted a strong oncolytic response, the presence of viral proteins (Sigma-3), a reduction in tumour cell proliferation (PCNA), and a loss of cytokeratins in PC-3M-Pro4luc2 tumours treated with *jin-3* reovirus. **G** Treatment with *jin-3* reovirus significantly reduced tumour volume in the PCa-15.01 PDX model (*n* = 10 per group; *p* < 0.01). **H** Significant tumour shrinkage upon intra-tumoural Jin 3 administration (tumour weight; *p* < 0.05). **I** In tumours treated with *jin-3* reovirus, viral *S4* RNA was detected (*p* < 0.0001). **J** Histological evaluation of *jin-3* reovirus-mediated anti-tumour effects indicated a loss of tissue architecture, the presence of viral proteins (Sigma-3), an induction of apoptosis (cleaved caspase-3), a reduction in proliferation (PCNA), and a loss of tumour-associated cytokeratins (PANKRT). Magnification is 63×, scale bar = 25 μm. Error bars indicated ± SEM, *p < 0.05, **p < 0.01, ***p < 0.001, ****p < 0.0001, two-way ANOVA and *t* tests.

Intra-tumoural administration of reovirus mutant *jin-3* reovirus in subcutaneously growing tumours from PC-3M-Pro4luc2 significantly decreased tumour burden (*p* = 0.0367) and diminished tumour volume (*p* = 0.06; Fig. 5A–D). Viral capsid protein RNA expression in tumour cells was significantly upregulated in *jin-3* reovirus-treated prostate tumours (S4 *p* < 0.0001; Fig. 5E). In line with the tumour regression data, histological analyses revealed that *jin-3* reovirus-treated tumours were depleted of proliferating cancer cells, their tissue architecture was lost and histological tumour markers significantly decreased. Conversely, high levels of viral Sigma-3 protein were detected in *jin-3* reovirus-treated tumours (Fig. 5F). Similar data were obtained with our novel prostate cancer PDX model PCa-15.01, in which intra-tumoural injection of *jin-3* reovirus significantly reduced tumour volume (*p* = 0.0072) and tumour weight (*p* = 0.0373; Fig. 5G, H). Likewise, *jin-3* reovirus-treated tumours displayed a strong induction of viral RNA S4 transcripts (*p* < 0.0001; Fig. 5I). Histological evaluation indicated a complete loss of tumour tissue architecture after *jin-3* reovirus treatment (Fig. 5J).

### *jin-3* reovirus induces determinants of immune modulation in human prostate cancer cells

**Fig. 6:**
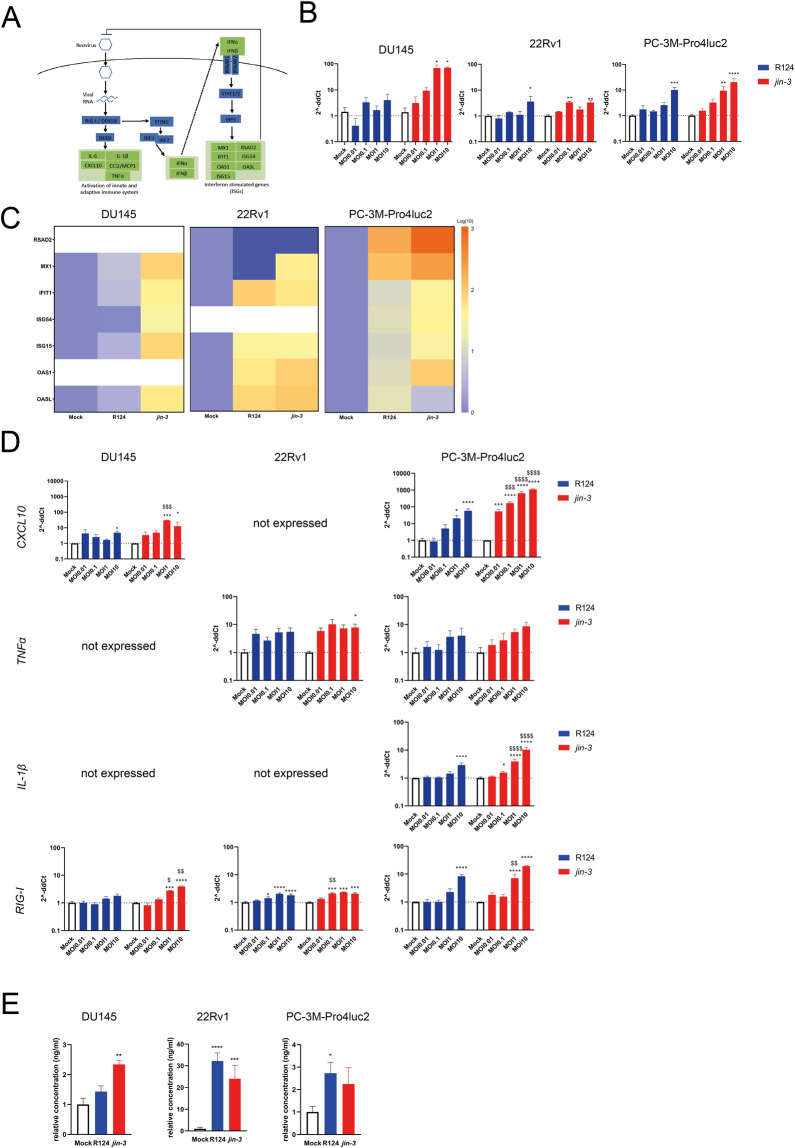
Immune modulatory response of R124 parental and *jin-3* mutant reovirus in prostate cancer cultures. **A** The role of the STING pathway and IFN signalling in recognising viral RNA and induction of anti-viral and anti-tumour immune responses. **B** Induction of *IFNβ* mRNA in prostate cancer cells after treatment with R124 or *jin-3* reovirus for 48 h. **C** Induction of interferon-stimulated genes (ISGs) in human prostate cancer cells after treatment with MOI10 of R124 or *jin-3* reovirus for 48 h (log-transformed). White boxes indicated that the gene was not expressed. **D** Induction of inflammatory cytokines gene expression of *CXCL10*, *TNFα*, and *IL-1β* and cytosolic RNA sensor *RIG-I* after treatment of human prostate cancer cells with reovirus. **E** HMGB1 protein release (danger-associated molecular pattern) by human prostate cancer cells after 48 h treatment with oncolytic reovirus. *p < 0.05, **p < 0.01, ***p < 0.001,****p < 0.0001, ^$^*p* < 0.05, ^$$^*p* < 0.01, ^$$$^*p* < 0.001, ^$$^ p < 0.0001. Mean ± standard error of the mean (SEM), *N* = 2. Two-way ANOVA.

Type I interferons and interferon-stimulated genes (ISGs) are of crucial importance in oncolytic virotherapy, as expression of ISGs associated with the sensitivity to oncolytic virotherapy [24, 25] (Fig. 6A). Exposure to *jin-3* reovirus resulted in the significant upregulation of *IFNβ* gene expression in human prostate cancer cells after 48 h (Fig. 6B). Compared to R124, exposure to *jin-*3 reovirus resulted in a significant stronger induction in IFNβ gene expression (^$^*p* < 0.05; Fig. 6B). Moreover, exposure of the cancer cells to *jin-3* reovirus resulted in a stronger induction of multiple ISGs compared to R124 (Fig. 6C). Furthermore, treatment with jin-3 reovirus induced a significant, dose-dependent induction of the inflammatory cytokines *CXCL10,* *TNFα*, and *IL-1β*. In addition, exposure to *jin-3* induced a significant stronger expression of *CXCL10* and *IL-1β* compared to R124 (^$$$^*p* < 0.001 and ^$$$$^*p* < 0.0001). Expression of *DDX58* that encodes for the cytosolic RNA sensor *RIG-I,* was also found to be significantly upregulated upon *jin-3* reovirus administration (Fig. 6D). In addition, shedding of HMGB1 protein, a well-established marker for immunogenic cell death, was observed after exposure of prostate cancer cells to *jin-3* reovirus [26] (Fig. 6E). Moreover, additional upstream targets of the anti-viral response were significantly affected by *jin-3* reovirus exposure, including *IFNAR1* and *IFNAR2* (Fig. S5).

## Discussion

Current clinical and preclinical evidence demonstrates that anti-tumour immune responses can eliminate existing malignant cells, resulting in the protection against tumour recurrence [27, 28]. However, the majority of prostate tumours does not adequately respond to immune therapy, which is mediated—among others—by the immunosuppressive tumour microenvironment, the low mutation burden resulting in limited expression of neoantigens, and immune evasion and exclusion processes in prostate tumours [4, 8, 29].

Oncolytic viruses are increasingly being exploited to counteract these immunosuppressive mechanisms by selectively killing cancer cells and, more importantly perhaps, by the initiation of anti-tumour immunity. Oncolytic viruses either have a natural preference to enter, replicate in, and/or kill cancer cells as opposed to normal cells or they are engineered to do so [30]. To achieve improved anti-tumour immunity in prostate cancer, the identification and clinical development of optimised oncolytic viruses, alone or combined with other treatment modalities, may be advantageous.

Here we report, for the first time, the use of *jin-3 r*eovirus in human prostate cancer models. Reovirus mutant *jin-3* reovirus induces potent direct anti-tumour effects in human prostate cancer. In ex vivo cultured human prostate cancer tissue slices and 2D/3D cell cultures, we found that *jin-3* reovirus is able to infect and replicate in the cancer cells. Moreover, *jin-3* reovirus exposure significantly induced the expression of ISGs and inflammatory cytokines. This is further substantiated by the release of immunogenic cell death marker HMGB1 after exposure of cancer cells to *jin-3* reovirus.

A number of preclinical studies have reported the anti-tumour effects of wild-type mammalian *Orthoreovirus* [31, 32]. In addition, clinical trials have revealed that wild-type reovirus has inherent oncolytic properties, is not associated with serious human disease, and has a favourable safely profile in cancer patients [11]. However, clinical trials have demonstrated that the clinical benefit of wild-type reovirus administration in prostate cancer patients is limited and so far phase II studies have revealed no survival benefit [11, 12, 32–36].

We previously described the isolation of reoviruses with an expanded tropism [10]. These so-called *jin* mutant reoviruses can also infect cancer cells independently of the canonical reovirus entry receptor JAM-A [10]. The potential oncolytic and immunomodulatory properties of these mutant reoviruses were not previously established in preclinical models of human prostate cancer. Here, we show that the reovirus mutant *jin-3* displays strong oncolytic and immunomodulatory properties and represents an interesting candidate oncolytic virus for the treatment of prostate cancers. We observed efficacious *jin-3* infection in the majority of ex vivo cultured prostate cancer tissue slices derived from PDXs and primary prostate cancer patient material. Moreover, *jin-3* induced significant tumour regression in various human prostate cancer xenografts in vivo as indicated by real-time optical imaging measurements (firefly luciferase2 expressing cancer cells) to determine the total tumour burden, changes in tumour volume, tumour weight, and reduction of pan-cytokeratin. In prolonged in vivo experiments and in a clinical setting, it will also be worthwhile to assess the effect of *jin-3 Reovirus* exposure on additional tumour markers in circulation, e.g., serum PSA. In this study, we observed that *jin-3* reovirus administration induces several ISGs and inflammatory cytokines. The *jin-3* reovirus-induced gene expression of key immunomodulators (activators of the adaptive and innate immune system) is further substantiated by the release of HMGB1 as one of the key DAMPs.

In conclusion, we describe the evaluation as an oncolytic agent of the mutant reovirus *jin-3* that displays strong oncolytic and immunomodulatory properties in state-of-the-art preclinical prostate cancer models, including patient-derived tumour slice models. Our findings support the notion that *jin-3* reovirus can be potentially exploited in strategic treatment combinations to with otherwise less efficacious immuno-oncological or chemotherapeutical approaches to achieve improved and durable anti-tumour responses.

## Supplementary information

Supplementary info

## References

[1] Bray F, Ferlay J, Soerjomataram I, Siegel RL, Torre LA, Jemal A (2018). Global cancer statistics 2018: GLOBOCAN estimates of incidence and mortality worldwide for 36 cancers in 185 countries. CA Cancer J Clin.

[2] Tang J, Shalabi A, Hubbard-Lucey VM (2018). Comprehensive analysis of the clinical immuno-oncology landscape. Ann Oncol.

[3] Bilusic M, Madan RA, Gulley JL (2017). Immunotherapy of prostate cancer: facts and hopes. Clin Cancer Res.

[4] Vitkin N, Nersesian S, Siemens DR, Koti M (2019). The tumor immune contexture of prostate cancer. Front Immunol.

[5] Davola ME, Mossman KL (2019). Oncolytic viruses: how “lytic” must they be for therapeutic efficacy?. Oncoimmunology.

[6] Errington F, Steele L, Prestwich R, Harrington KJ, Pandha HS, Vidal L (2008). Reovirus activates human dendritic cells to promote innate antitumor immunity. J Immunol.

[7] Prestwich RJ, Errington F, Steele LP, Ilett EJ, Morgan RS, Harrington KJ (2009). Reciprocal human dendritic cell-natural killer cell interactions induce antitumor activity following tumor cell infection by oncolytic reovirus. J Immunol.

[8] Lee P, Gujar S (2018). Potentiating prostate cancer immunotherapy with oncolytic viruses. Nat Rev Urol.

[9] Kemp V, Lamfers MLM, van der Pluijm G, van den Hoogen BG, Hoeben RC (2020). Developing oncolytic viruses for clinical use: A consortium approach. Cytokine Growth Factor Rev.

[10] van den Wollenberg DJ, Dautzenberg IJ, van den Hengel SK, Cramer SJ, de Groot RJ, Hoeben RC (2012). Isolation of reovirus T3D mutants capable of infecting human tumor cells independent of junction adhesion molecule-A. PLoS ONE.

[11] Vidal L, Pandha HS, Yap TA, White CL, Twigger K, Vile RG (2008). A phase I study of intravenous oncolytic reovirus type 3 Dearing in patients with advanced cancer. Clin Cancer Res.

[12] Thirukkumaran CM, Nodwell MJ, Hirasawa K, Shi ZQ, Diaz R, Luider J (2010). Oncolytic viral therapy for prostate cancer: efficacy of reovirus as a biological therapeutic. Cancer Res.

[13] Barton ES, Forrest JC, Connolly JL, Chappell JD, Liu Y, Schnell FJ (2001). Junction adhesion molecule is a receptor for reovirus. Cell.

[14] Zhao C, Lu F, Chen H, Zhao X, Sun J, Chen H (2014). Dysregulation of JAM-A plays an important role in human tumor progression. Int J Clin Exp Pathol.

[15] van de Merbel AF, van der Horst G, van der Pluijm G (2021). Patient-derived tumour models for personalized therapeutics in urological cancers. Nat Rev Urol.

[16] Dautzenberg IJ, van den Wollenberg DJ, van den Hengel SK, Limpens RW, Barcena M, Koster AJ (2014). Mammalian orthoreovirus T3D infects U-118 MG cell spheroids independent of junction adhesion molecule-A. Gene Ther.

[17] Gao D, Vela I, Sboner A, Iaquinta PJ, Karthaus WR, Gopalan A (2014). Organoid cultures derived from patients with advanced prostate cancer. Cell.

[18] Drost J, Karthaus WR, Gao D, Driehuis E, Sawyers CL, Chen Y (2016). Organoid culture systems for prostate epithelial and cancer tissue. Nat Protoc.

[19] van de Merbel AF, van der Horst G, van der Mark MH, van Uhm JIM, van Gennep EJ, Kloen P (2018). An ex vivo tissue culture model for the assessment of individualized drug responses in prostate and bladder cancer. Front Oncol.

[20] Navone NM, van Weerden WM, Vessella RL, Williams ED, Wang Y, Isaacs JT (2018). Movember GAP1 PDX project: An international collection of serially transplantable prostate cancer patient-derived xenograft (PDX) models. Prostate.

[21] van den Hoogen C, van der Horst G, Cheung H, Buijs JT, Lippitt JM, Guzman-Ramirez N (2010). High aldehyde dehydrogenase activity identifies tumor-initiating and metastasis-initiating cells in human prostate cancer. Cancer Res.

[22] van Weerden WM, de Ridder CM, Verdaasdonk CL, Romijn JC, van der Kwast TH, Schroder FH (1996). Development of seven new human prostate tumor xenograft models and their histopathological characterization. Am J Pathol.

[23] Marques RB, Dits NF, Erkens-Schulze S, van Weerden WM, Jenster G (2010). Bypass mechanisms of the androgen receptor pathway in therapy-resistant prostate cancer cell models. PLoS ONE.

[24] Matveeva OV, Chumakov PM (2018). Defects in interferon pathways as potential biomarkers of sensitivity to oncolytic viruses. Rev Med Virol.

[25] Kurokawa C, Iankov ID, Anderson SK, Aderca I, Leontovich AA, Maurer MJ (2018). Constitutive interferon pathway activation in tumors as an efficacy determinant following oncolytic virotherapy. J Natl Cancer Inst.

[26] Kepp O, Senovilla L, Vitale I, Vacchelli E, Adjemian S, Agostinis P (2014). Consensus guidelines for the detection of immunogenic cell death. Oncoimmunology.

[27] Waldman AD, Fritz JM, Lenardo MJ (2020). A guide to cancer immunotherapy: from T cell basic science to clinical practice. Nat Rev Immunol..

[28] Garner H, de Visser KE (2020). Immune crosstalk in cancer progression and metastatic spread: a complex conversation. Nat Rev Immunol.

[29] Silvestri I, Cattarino S, Aglianò AM, Collalti G, Sciarra A (2015). Beyond the immune suppression: the immunotherapy in prostate cancer. Biomed Res Int.

[30] Kemp V, Lamfers MLM, van der Pluijm G, van den Hoogen BG, Hoeben RC (2020). Developing oncolytic viruses for clinical use: A consortium approach. Cytokine Growth Factor Rev.

[31] Thirukkumaran CM, Shi ZQ, Luider J, Kopciuk K, Gao H, Bahlis N (2012). Reovirus as a viable therapeutic option for the treatment of multiple myeloma. Clin Cancer Res.

[32] Heinemann L, Simpson GR, Boxall A, Kottke T, Relph KL, Vile R (2011). Synergistic effects of oncolytic reovirus and docetaxel chemotherapy in prostate cancer. BMC Cancer.

[33] Gujar SA, Pan DA, Marcato P, Garant KA, Lee PW (2011). Oncolytic virus-initiated protective immunity against prostate cancer. Mol Ther.

[34] Comins C, Spicer J, Protheroe A, Roulstone V, Twigger K, White CM (2010). REO-10: a phase I study of intravenous reovirus and docetaxel in patients with advanced cancer. Clin Cancer Res.

[35] Eigl BJ, Chi K, Tu D, Hotte SJ, Winquist E, Booth CM (2018). A randomized phase II study of pelareorep and docetaxel or docetaxel alone in men with metastatic castration resistant prostate cancer: CCTG study IND 209. Oncotarget.

[36] Berkeley RA, Steele LP, Mulder AA, van den Wollenberg DJM, Kottke TJ, Thompson J (2018). Antibody-neutralized reovirus is effective in oncolytic virotherapy. Cancer Immunol Res.

