# Supplementary material for: Reovirus mutant jin-3 exhibits lytic and immune-stimulatory effects in preclinical human prostate cancer models
Source: Cancer Gene Ther. 2021 Jun 16;29(6):793–802. doi: 10.1038/s41417-021-00360-2 (PMC9209329; doi:10.1038/s41417-021-00360-2)
Supplement: Supplementary file 1 — Supplementary info [file 41417_2021_360_MOESM1_ESM.docx]

**Table S1: Cell culture media**

| **Cell line** | **RRID** | **Medium** | **Supplier** | **Supplements** |
| --- | --- | --- | --- | --- |
| PC-3M-Pro4luc2 |  | Dulbecco’s Modified Eagle medium (DMEM) | Life technologies, Gibco, 31966-021 | 10% FCII (Hyclone), 100 units/ml penicillin, 50 μg/ml streptomycin  800 µg/mL of G-418 |
| DU145 | ATCC Cat# HTB-81, RRID:CVCL_0105 | RPMI 1640 | Lonza, BE12-167F | 10% FBS, 100 units/ml penicillin,  50 μg/ml streptomycin, GlutaMAX |
| 22Rv1 | ATCC Cat# CRL-2505, RRID:CVCL_1045 | RPMI 1640 | Lonza, BE12-167F | 10% FBS, 100 units/ml penicillin,  50 μg/ml streptomycin, GlutaMAX |

**Table S2: Antibodies used for immunofluorescence stainings and FACS analyses**

| *Target* | *Species* | *Supplier* | *Dilution* | *Assay* |
| --- | --- | --- | --- | --- |
| 4F2 against reovirus σ3 | Mouse | Antibody was developed by T.S. Dermody ^36^; purchased at the Developmental Studies Hybridoma Bank developed under the auspices of the NICHD and maintained by The University of Iowa, Department of Biology, Iowa City, IA52242 | 1:200 | ICC, IF |
| Pan cytokeratin | Rabbit | Abcam ab217916 | 1:500 | IF |
| Collagen type I | Goat | Southern Biotech No 1310-01 | 1:1000 | IF |
| AMACR | Rabbit | Atlas antibody HPA019527 | 1:5000 | IF |
| PCNA | Mouse | Sigma Aldrich P8825 | 1:2000 | IF |
| Cleaved caspase-3 | Rabbit | Cell Signaling 9661L | 1:500 | IF |
| JAM-A | Mouse | Abnova H00050848-M01 | 1:250 | IF |
| JAM-A | Mouse | Abcam ab17261 | 1:200 | FACS |
| AR | Rabbit | Cell Signaling #5153 | 1:500 | IF |
| Donkey anti-mouse Alexa Fluor 488 | Donkey | Life Technologies A-21202 |  | ICC, IF, FACS, |
| Donkey anti-rabbit Alexa Fluor 488 | Donkey | Life Technologies A-21206 |  | IF |
| Donkey anti-mouse Alexa Fluor 555 | Donkey | Life Technologies A-31570 |  | IF |
| Donkey anti-rabbit Alexa Fluor 555 | Donkey | Life Technologies A-31572 |  | IF |
| Donkey anti-rabbit Alexa Fluor 647 |  | Life Technologies A-21447 |  | IF |

**Table S3: Characteristics of newly generated patient-derived xenograft models and primary prostate cancer biopsies**

| Novel established models and patient biopsies used for *ex vivo* cultures | | | | | |  |
| --- | --- | --- | --- | --- | --- | --- |
| Sample name | Source | TNM stage | Gleason Grade | PSA (ug/l) | Treatment history | |
| PCa-15.01 | Prostatectomy (hormone naïve) | T3NxM+ | 4+5 | >5000 | No previous treatment | |
| NM60 | Needle biopsy liver metastasis (CRPC) | Not applicable | Not applicable | 81.0 | Zoladex, Docetaxel, Abiraterone, radiotherapy, Cabazitaxel, Carboplatin, Olaparib | |
| NM72 | Needle biopsy liver metastasis (CRPC) | M1c | Not applicable | 51.0 | Prostatectomy, Docetaxel, Abiraterone, Cabazitaxel, Olaparib, Cabazitaxel/Carboplatin | |
| NM78 | Prostate cancer bone metastases | M1b | Not applicable | 470.0 | EBRT, Androgen deprivation (Eligard), Enzalutamide, Abiraterone | |
| Patient #1 | Transurethral resection | T4N+M+ | 4+4 | 52.5 | EBRT, LHRH agonist, Enzalutamide | |
| Patient #2 | Transurethral resection | T3bNxMx | 3+4 | 7.5 | LHRH agonist | |
| Patient #3 | Transurethral resection | T3bN0Mx | 4+4 | 15.0 | Androgen deprivation | |
| Patient #4 | Transurethral resection | T3bN+M+ | 5+5 | 1.58 | LHRH agonist, Enzalutamide | |
| Patient #6 | Transurethral resection | T4N1M1b | 5+5 | 29.36 | Bicalutamide, Zoladex | |
| Patient #7 | Transurethral resection | T3aN0M0 | 4+5 | 14.23 | EBRT, Bicalutamide, Goserelin | |
| Patient #8 (NM91) | Prostate cancer bone metastases | M1b | Not applicable | 21.0 | Prostatectomy, EBRT, Docetaxel, Enzalutamide, Talazoparib, ongoing bone resection | |

**Table S4: Characteristics of previously established three-dimensional cultures and patient-derived xenograft lines**

| Previously established models | | | | | |
| --- | --- | --- | --- | --- | --- |
| Tumour model | Source | Treatment history | Clinical status | Diagnosis | References |
| MSK-PCa1 | L2 vertebral body | Androgen-deprivation therapy, bicalutamide | mCRPC | Intraductal carcinoma | ^17^ |
| MSK-PCa2 | Acetabulum | Androgen-deprivation therapy, bicalutamide | mCRPC | Adenocarcinoma | ^17^ |
| PC82 | Radical prostatectomy | No previous treatment | hormone naïve | Adenocarcinoma | ^20, 22^ |
| PC295 | Lymph node resection | None | hormone naïve | Adenocarcinoma | ^20, 22^ |
| PC310 | Radical prostatectomy | None | hormone naïve | Adenocarcinoma | ^20, 22^ |
| PC324 | Transurethral resection | Bilateral orchiectomy | mCRPC | Adenocarcinoma and neuroendocrine differentiation | ^20, 22^ |
| PC339C | Transurethral resection | LHRH agonist | mCRPC | Adenocarcinoma | ^20, 22^ |
| PC346C | Transurethral resection | Anti-androgen | Hormone responsive | Adenocarcinoma | ^22, 23^ |
| PC374 | Scrotal skin metastasis biopsy | Androgen deprivation, radiotherapy, anti-androgen | mCRPC | Adenocarcinoma | ^20, 22^ |

**Table S5: Primer sequences used for RTqPCR**

| *Target* | *Primer sequence* |
| --- | --- |
| *Mycoplasma A1* | tgcaccatctgtcactctgttaacctc |
| *Mycoplasma A2* | *gggagcaaacaggattagataccct* |
| *Human GAPDH FW* | gacagtcagccgcatcttc |
| *Human GAPDH RV* | gcaacaatatccactttaccagag |
| *Universal GAPDH FW* | ccatggagaaggctgggg |
| *Universal GAPDH RV* | caaagttgtcatggatgacc |
| *MX1 FW* | tcagcacctgatggccta |
| *MX1 RV* | aaagggatgtggctggagat |
| *OASL FW* | ttgctatgacaacagggagaac |
| *OASL RV* | ctgtcaagtggatgtctcgtg |
| *DDX58 FW* | tgtgggcaatgtcatcaaaa |
| *DDX58 RV* | gaagcacttgctacctcttgc |
| *CXCL10 FW* | gaaagcagttagcaaaggaaaggt |
| *CXCL10 RV* | gacatatactccatgtagggaagtga |
| *RSAD2 FW* | tgcttttgcttaaggaagctg |
| *RSAD2 RV* | aggtattctccccggtcttg |
| *ISG54 FW* | atataggtctcttcagcatttattggt |
| *ISG54 RV* | caaggaattcttattgttctcactca |
| *TNFa FW* | cagcctcttctccttcctgat |
| *TNFa RV* | gccagagggctgattagaga |
| *IL1B FW* | tacctgtcctgcgtgttgaa |
| *IL1B RV* | tctttgggtaatttttgggatct |
| *IFNAR1 FW* | atttacaccatttcgcaaagc |
| *IFNAR1 RV* | cactattgccttatcttcagcttcta |
| *IFNAR2 FW* | tagcctccccaaagtcttga |
| *IFNAR2 RV* | aaatgacctccaccatatcca |
| *ISG15 FW* | gcgaactcatctttgccagta |
| *ISG15 RV* | ccagcatcttcaccgtcag |
| *IFIT1 FW* | gcctaatttacagcaaccatga |
| *IFIT1 RV* | caaggaattcttattgttctcactca |
| *IFNB FW* | ctttgctattttcagacaagattca |
| *IFNB RV* | gccaggaggttctcaacaat |
| *S4Q FW* | cgcttttgaaggtcgtgtatca |
| *S4Q RV* | ctggctgtgctgagattgtttt |

**
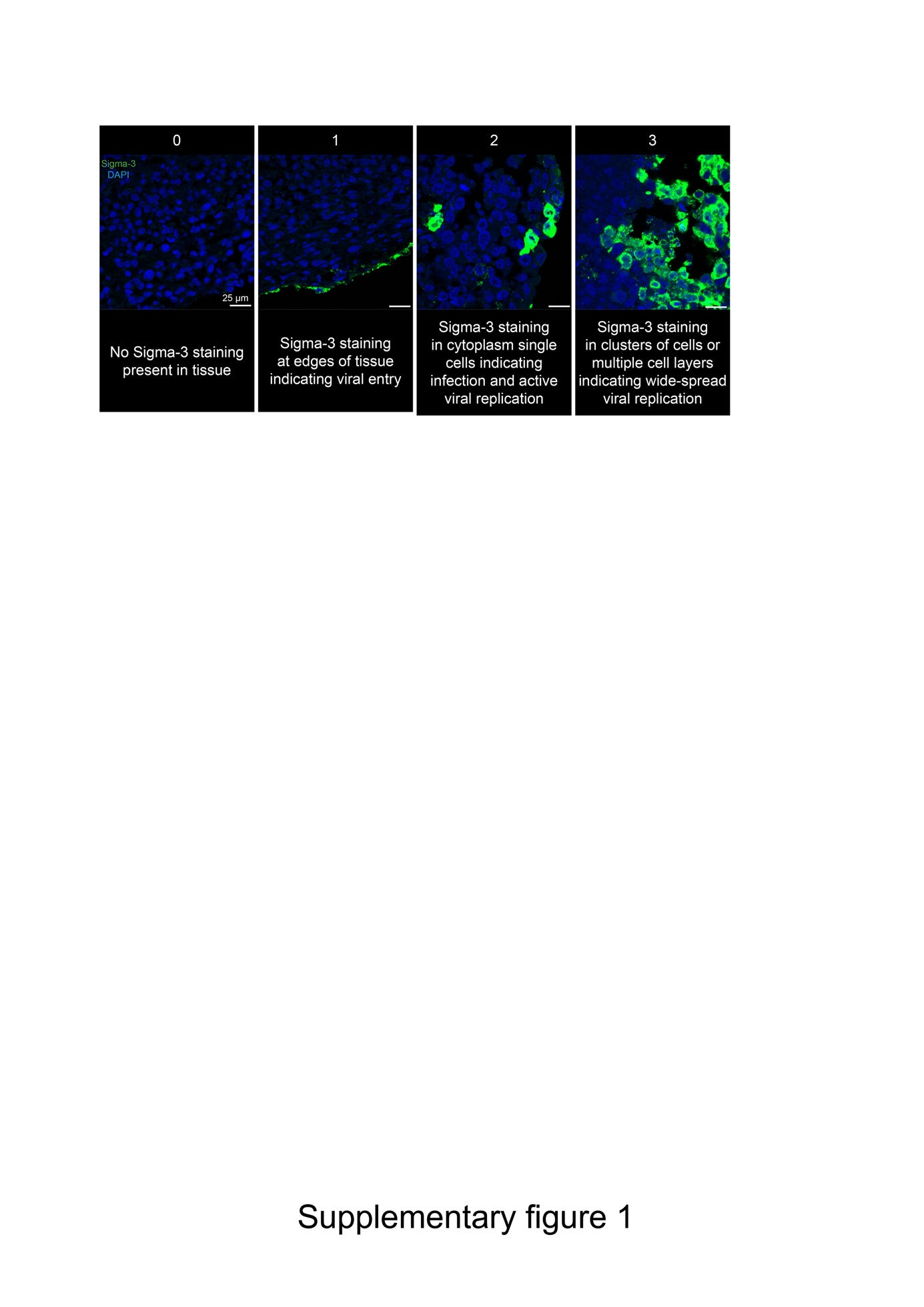
**

**Figure S1: Sigma-3 scoring system**

Prostate cancer tissue slices exposed to reovirus were stained with an antibody directed against viral protein Sigma-3 and scored according to Sigma-3 pattern ranging from 0 to 3.

**
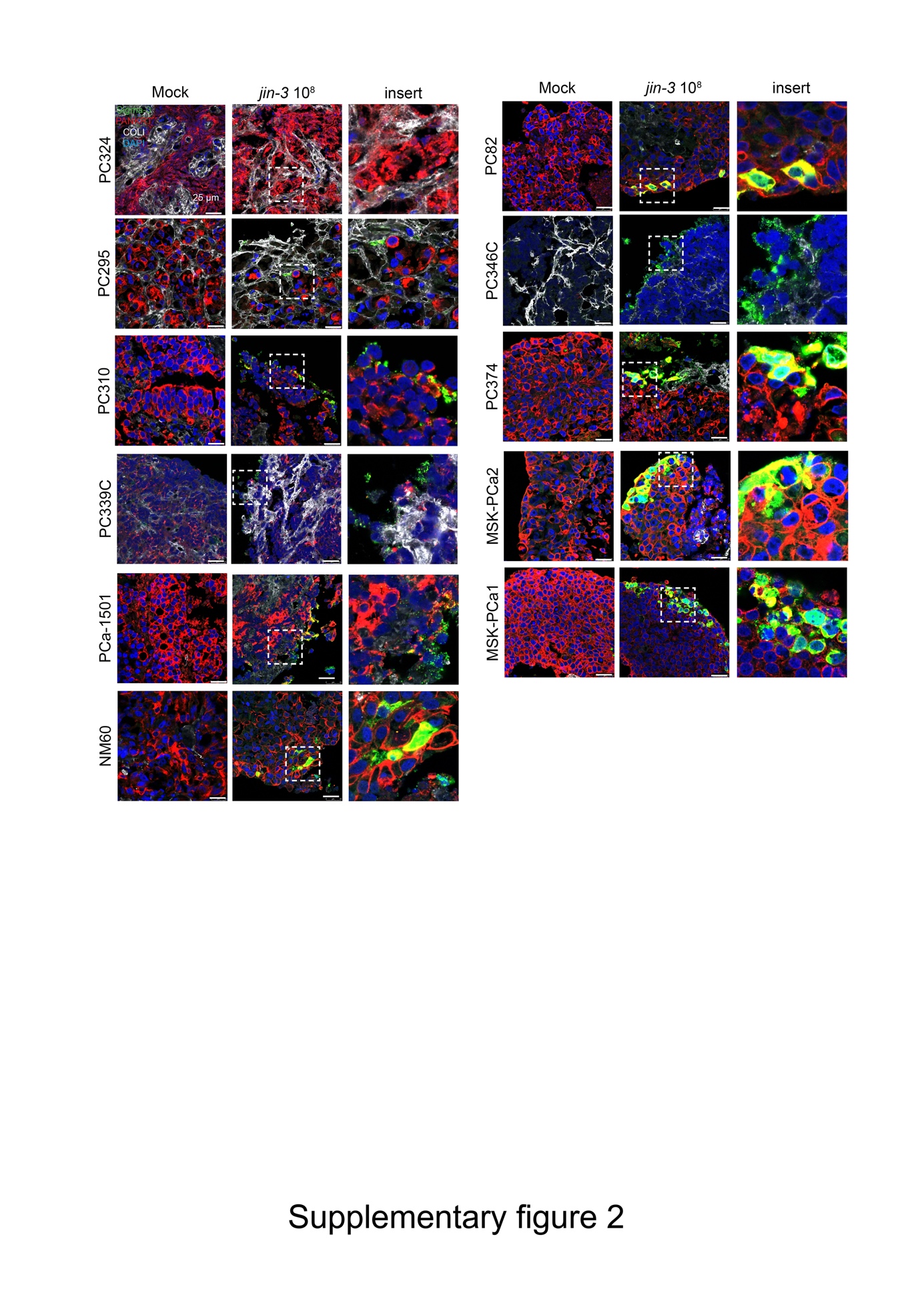
**

**Figure S2: Sigma-3 staining in prostate cancer tissue slices derived from PDX models upon reovirus exposure** Sigma-3 (green), pan-cytokeratin (red), type I collagen (white), DAPI (blue) staining in human prostate cancer tissue slices derived from PDX models. Prostate cancer tissue slices were *ex vivo* exposed to 10^8^ pfu/ml reovirus for 3 days. Magnification is 63x, scale bar = 25 μm

**
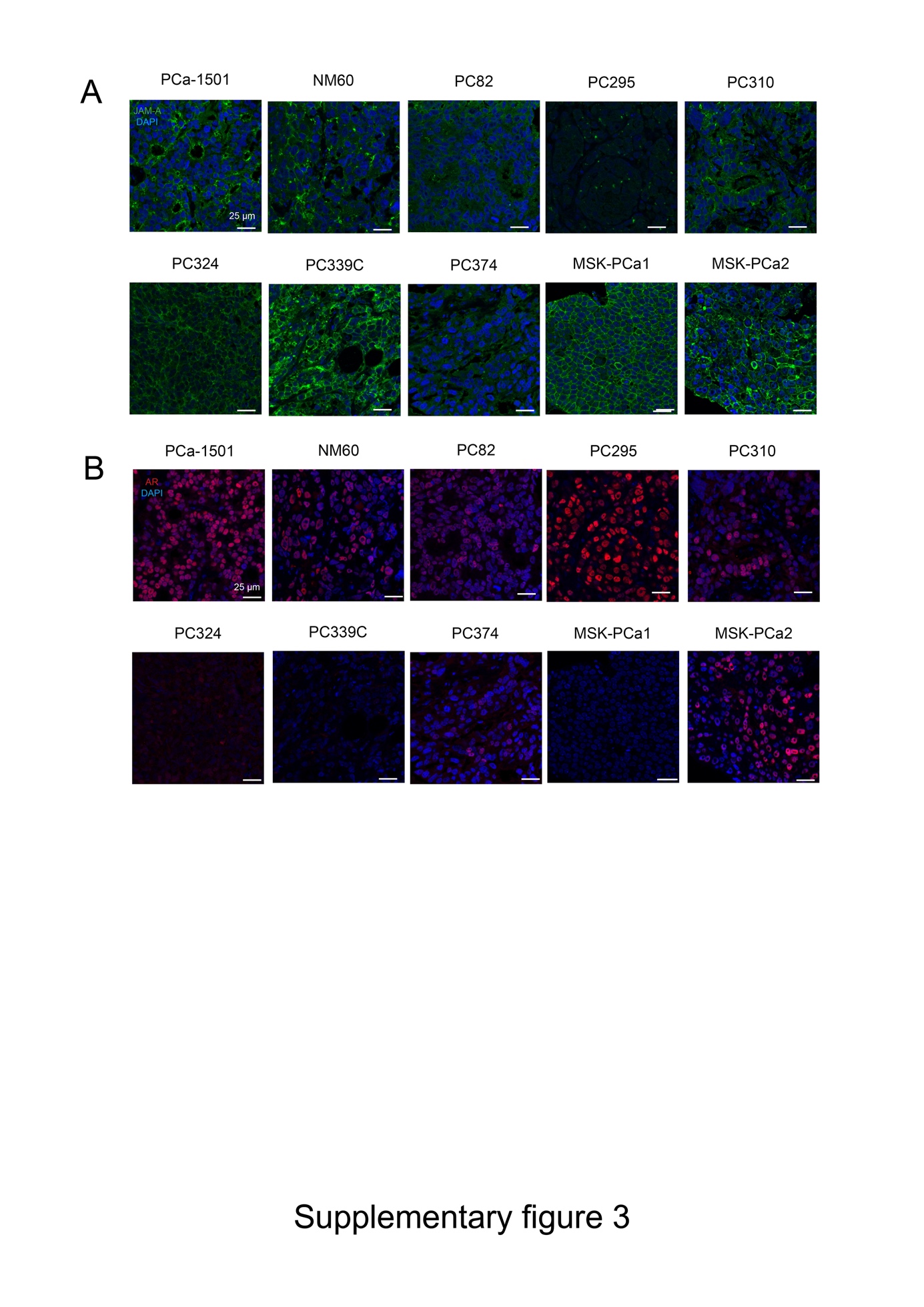
**

**Figure S3: JAM-A and AR expression in prostate cancer patient-derived xenograft (PDX) models**

Immunolocalisation of reovirus entry receptor junction adhesion molecule-A (JAM-A) (A) and the androgen receptor (AR) (B) in a broad spectrum of human prostate cancer PDX tissues (green). Magnification is 63x, scale bar = 25 μm


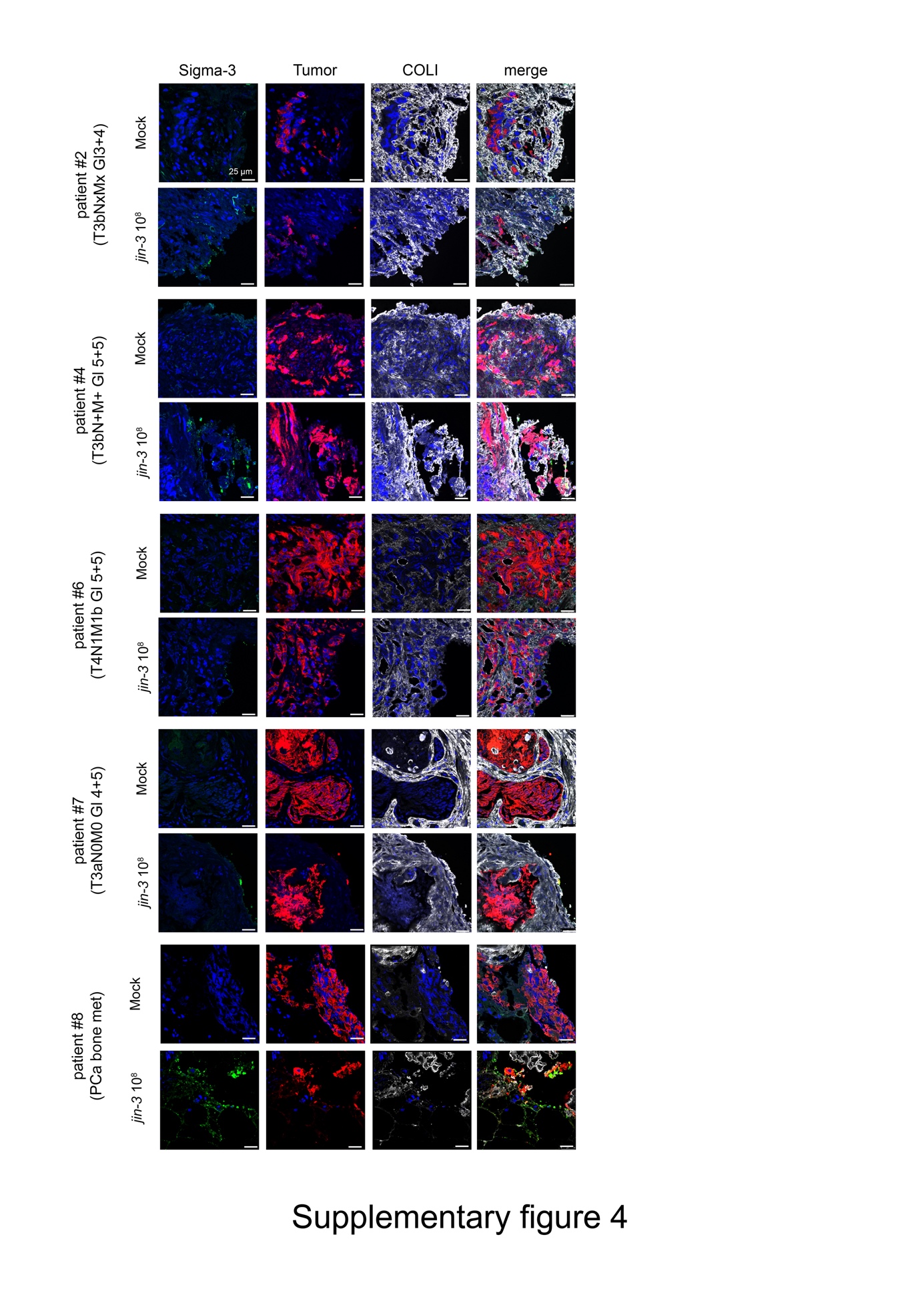


**Figure S4: Sigma-3 staining in primary prostate cancer tissue slices derived from transurethral resection of the prostate upon reovirus exposure**

Sigma-3 (viral protein, green), pan-cytokeratin or AMACR (tumour cells, red), type I collagen fibres (white), DAPI (blue, nuclei) stainings in prostate cancer tissue slices derived from transurethral resection of the prostate primary samples. Prostate cancer tissue slices were *ex vivo* exposed to 10^8^ pfu/ml reovirus for 3 days. Magnification is 63x, scale bar = 25 μm


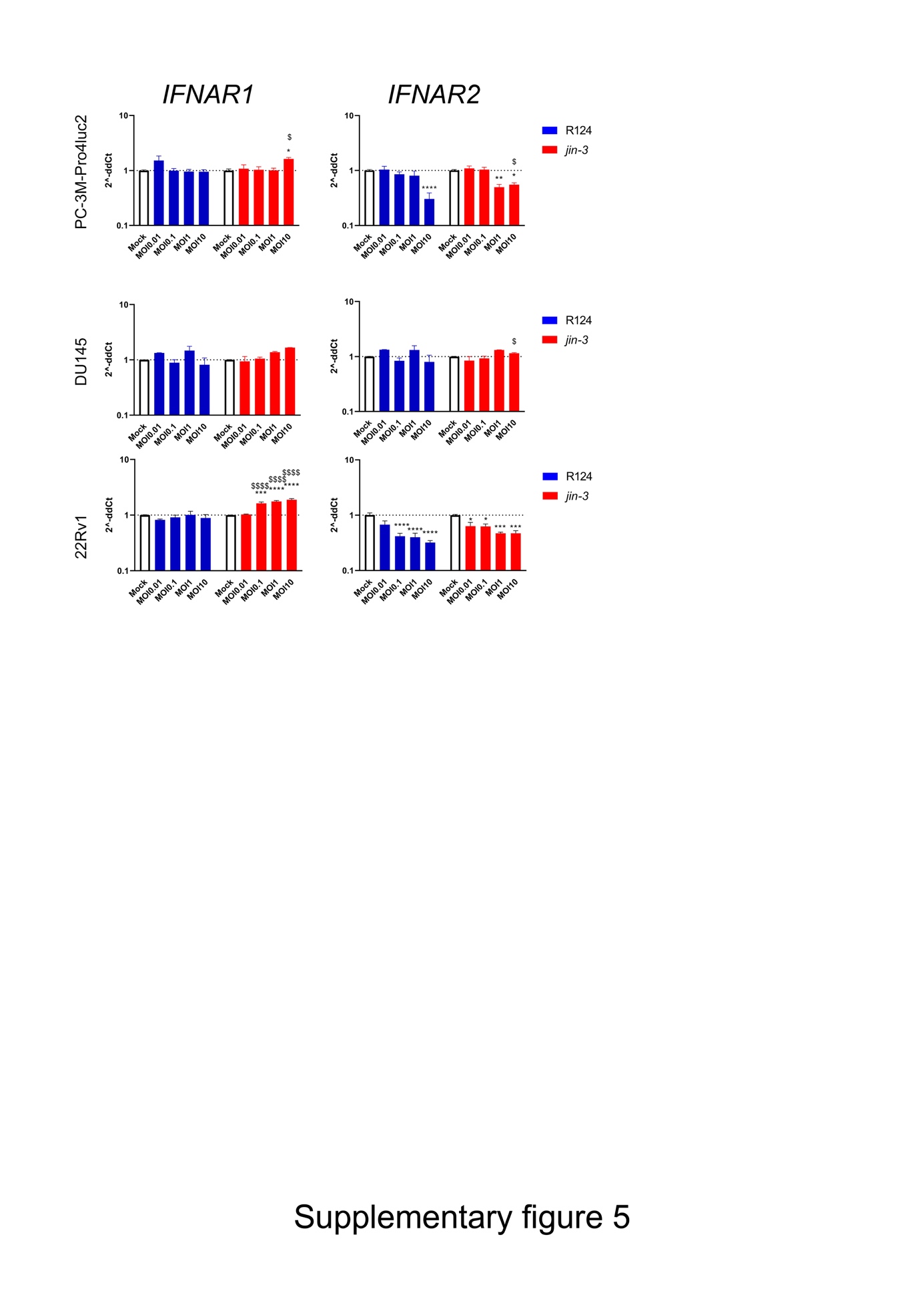


**Figure S5: Changes in IFNAR1, IFNAR2 mRNA expression upon reovirus exposure**

Infection with *jin-3* changed mRNA expression of IRNAR1, IFNAR2 after 48h in human prostate cancer cells. * p<0.05, ** p<0.01, *** p<0.001, **** p<0.0001, $ p<0.05, $$ p<0.01, $$$ p<0.001, $$$$ p<0.0001. Mean +/- standard error of the mean (SEM), N=2. Two-way ANOVA. MOI = multiplicity of infection

**Supplementary information**

*Sample size*

Sample size was chosen according to expected differences in the groups according to the following formula: n=2∙(Z_((1-α/2))+Z_((1-β)) )^2/∆^2 ∆=(μ1-μ2)/σ.

(n= sample size , Z_((1-α/2)) = percentage points of the normal distribution for the statistical significance level, Z_((1-β))= percentage points of the normal distribution for the power, Δ= standardized difference, μ=mean, σ= standard deviation).

For calculation of the sample size for the androgen independent in vivo model (PC-3M-Pro4luc) we have used the relative light units measured with bioluminescent imaging. With expected mean value of the tumour burden is 3.5*10^9 RLU with a standard deviation of 1 *10^9. Expected difference is -45%. Power: 80%, significance level: 5%

For the androgen dependent in vivo model (PCa-15.01): we have calculated the sample size using the expected tumour volume. The expected mean value of the tumour burden is 1.5cm^3 with a standard deviation of 0.5cm^3. Expected difference is -45%. Power: 80%, significance level: 5%

*Exclusion criteria*

Pre-established exclusion criteria for the preclinical *in vivo* models: when tumour does not develop, the mouse will be excluded from the experiment. Further exclusion criteria for analysis of the data: when a mouse will reach humane endpoints before the end of the experiment, this mouse will be excluded from analysis.

No mice were excluded based on these criteria in our reported experiments.
